# Alpha-Methyldopa-Induced Autoimmune Hemolytic Anemia in the Third Trimester of Pregnancy

**DOI:** 10.1155/2013/150278

**Published:** 2013-09-23

**Authors:** Charalampos Grigoriadis, Aliki Tympa, Angelos Liapis, Dimitrios Hassiakos, Panagiotis Bakas

**Affiliations:** ^1^2nd Department of Obstetrics and Gynecology, Medical School of Athens, Aretaieion University Hospital, Vas. Sofias Avenue, 11528 Athens, Greece; ^2^1st Department of Anesthesiology, Medical School of Athens, Aretaieion University Hospital, Vas. Sofias Avenue, 11528 Athens, Greece

## Abstract

Alpha-methyldopa has been demonstrated to be safe for use during pregnancy and is now used to treat gestational hypertension. In pregnancy, alpha-methyldopa-induced autoimmune hemolytic anemia does not have typical features and the severity of symptoms ranges from mild fatigue to dyspnea, respiratory failure, and death if left untreated. A case of alpha-methyldopa-induced autoimmune hemolytic anemia in a 36-year-old gravida 2, para 1 woman at 37^+6^ weeks of gestation is reported herein along with the differential diagnostic procedure and the potential risks to the mother and the fetus.

## 1. Introduction

Drug-induced autoimmune hemolytic anemia (DIAIHA) can be underrecognized in pregnancy due to the physiological dilutional anemia observed. Autoimmune hemolytic anemia (AIHA) accounts for 0.8–3 per 100 000 cases of anemia [1], and DIAIHA accounts for 1 case per 1 000 000 [2]. Alpha-methyldopa has been demonstrated to be safe for use during pregnancy and is now used to treat gestational hypertension. Nonetheless, it has been speculated to be one of the first causes [3] of drug-induced warm autoimmune hemolytic anemia (WAIHA), which is now rarely encountered due to a decline in methyldopa use in the general population.

A case of alpha-methyldopa-induced autoimmune hemolytic anemia of pregnancy is reported herein along with the major problems in differential diagnosis and the risks posed to both the mother and the fetus.

## 2. Case Presentation

A 36-year-old gravida 2, para 1 woman at 37^+6^ weeks of gestation presented to the obstetric unit for elective cesarean delivery due to a previous cesarean section for breech presentation. Medical history included *β*-Mediterranean thalassemia trait and gestational hypertension (ranging between 146/90 and 170/100 mmHg) under alpha-methyldopa 500 mg twice daily since the 24th week of gestation. Abnormal laboratory findings included microcytic, hypochromic, and anisocytic anemia, hemoglobin 8.09 g/dL (normal range: 12–16 g/dL), lymphocytes 14.2 × 10^3^ *μ*L^−1^ (normal range: 20–51 × 10^3^ *μ*L^−1^), erythrocyte sedimentation rate 33 mm/h (normal range: <25 mm/h), and reticulocyte production index 3.1 (normal range: 0.2–2). Physical examination revealed easy fatigability over the previous few weeks, tachycardia (115 beats per minute), and pallor.

A healthy male infant with Apgar scores of 9 and 10 at the first and the fifth minutes, respectively, was born by cesarean section under combined spinal-epidural anesthesia. Physical examination of the newborn was unremarkable with no signs of hemolysis throughout its stay in the fetal medicine unit. In the same evening, maternal hemoglobin levels decreased to 7 g/dL with no signs of hemorrhage from the surgical field and reached 6.1 g/dL over the next three days. Direct antiglobulin (DAT) test was strongly positive (3+) for IgGs. The baby's blood group type was O Rh(−), and the mother's blood group type was AB Rh(+). Serum liver, renal functions, coagulation studies, and urine analysis were normal. Alpha-methyldopa was discontinued immediately upon positive DAT and was replaced by intravenous hydralazine. Hemoglobin levels returned to the pregestational levels (10 g/dL) one month after alpha-methyldopa discontinuation, still with a positive DAT.

## 3. Discussion

Alpha-methyldopa-induced AIHA is rare in pregnancy with only few cases published. Hemolysis due to alpha-methyldopa occurs via autoantibodies targeted against red blood cells [3]. Alpha-methyldopa causes the production of such antibodies in about 15% of patients receiving the drug, with 0.5%–1% developing hemolytic anemia [4]. Alpha-methyldopa-induced hemolysis is categorized as true autoimmune and drug-independent antibody [5], meaning that the antibody is in vitro reactive, even in the absence of the drug. In that case, hemolysis is always extravascular in type and IgG-mediated, has a slow onset, can occur by three months after starting alpha-methyldopa, and may take years until a negative DAT is achieved.

Alpha-methyldopa-induced AIHA does not have typical features, and the severity of symptoms ranges from mild fatigue [2] to dyspnea, respiratory failure [5], and death [3] if left untreated, with hemoglobin levels reaching as low as 5 g/dL [5]. Concerning methyldopa-induced atypical presentations, Ozdemir et al. [6] reported a case of a newborn with unconjugated hyperbilirubinemia and positive DAT upon alpha-methyldopa administration to the mother during pregnancy.

Alpha-methyldopa-induced AIHA is uncommon and in the laboratory setting yields similar findings with WAIHA: positive DAT, positive indirect antiglobulin test, and positive elution; however, the mechanism of hemolysis is more complex and heterogeneous with immune dysregulation, autoantibodies via molecular mimicry, and drug adsorption causing altered red blood cell membrane antigens [5]. WAIHAs may occur as idiopathic or may be associated with malignancies, infections, collagen disorders, and blood transfusions [7]. As far as the obstetric population is concerned, AIHA may occur in the first or the third trimester of pregnancy [7, 8]. When it comes to pregnancy, DIAIHA does not come under suspicion soon enough, as the patient already has progressive anemia and no other signs of hemolysis. Investigation usually starts upon presentation to the obstetric unit and routine preoperative laboratory testing. 

Distinguishing DIAIHA from WAIHA can be difficult. The clinician should have in mind that WAIHAs may be associated with platelet function disorders and pancytopenias. In the perioperative setting, it appears that regional anesthetic techniques can be applied only upon exclusion of platelet function disorders. In the present case, the medical history raised suspicion of alpha-methyldopa-induced AIHA, and coagulation parameters let us opt for combined spinal epidural anesthesia that would be also beneficial for hypertension. 

Definitely distingushing WAIHAs from DIAHAs requires complex and time-consuming testing, performed by hematology referral centers. In the obstetric setting, this is not always possible, given the urgency of the procedure in some cases. Drug history remains crucial for the investigation, and drug cessation followed by hemolytic anemia resolution remains the only reliable final confirmation of DIAIHA.

## Abbreviations

DIAIHA: Drug-induced autoimmune hemolytic anemia
AIHA: Autoimmune hemolytic anemia
WAIHA: Warm autoimmune hemolytic anemia.
